# Electrocardiographic Time-Intervals Waveforms as Potential Predictors for Severe Acute Kidney Injury in Critically Ill Patients

**DOI:** 10.3390/jcm12020700

**Published:** 2023-01-16

**Authors:** Francesco Corradi, Gregorio Santori, Claudia Brusasco, Chiara Robba, Adrian Wong, Pierpaolo Di Nicolò, Ludovica Tecchi, Federico Dazzi, Erika Taddei, Alessandro Isirdi, Federico Coccolini, Francesco Forfori, Guido Tavazzi, on behalf of UCARE Research Group

**Affiliations:** 1 Department of Surgical, Medical, Molecular Pathology and Critical Care Medicine, University of Pisa, 56126 Pisa, Italy; 2 Azienda Ospedaliero Universitaria Pisana, Via Paradisa, 2, 56124 Pisa, Italy; 3 Department of Surgical Sciences and Integrated Diagnostics (DISC), University of Genoa, 16126 Genoa, Italy; 4 Anesthesia and Intensive Care Unit, E.O. Ospedali Galliera, 16128 Genoa, Italy; 5 Anesthesia and Intensive Care, Policlinico San Martino, IRCCS for Oncology and Neuroscience, 16126 Genoa, Italy; 6 Department of Critical Care, King’s College Hospital, London SE5 9RS, UK; 7 Nephrology and Dialysis Unit, “S. Maria della Scaletta” Hospital, 40026 Imola, Italy; 8 Department of Clinical Surgical, Diagnostic and Pediatric Sciences, University of Pavia, 27100 Pavia, Italy; 9 Anaesthesia, Intensive Care and Pain Therapy, Fondazione IRCCS Policlinico San Matteo, 27100 Pavia, Italy

**Keywords:** splanchnic congestion, hypoperfusion, renal ultrasonography, renal Doppler resistive index, venous impedance index, acute kidney disease

## Abstract

Background: Acute kidney injury (AKI) is common in critically ill patients admitted to intensive care units (ICU) and is frequently associated with poorer outcomes. Hence, if an indicator is available for predicting severe AKI within the first few hours of admission, management strategies can be put into place to improve outcomes. Materials and methods: This was a prospective, observational study, involving 63 critically ill patients, that aimed to explore the diagnostic accuracy of different Doppler parameters in predicting AKI in critically ill patients from a mixed ICU. Participants were enrolled at ICU admission. All underwent ultrasonographic examinations and hemodynamic assessment. Renal Doppler resistive index (RDRI), venous impedance index (VII), arterial systolic time intervals (a-STI), and venous systolic time intervals (v-STI) were measured within 2 h from ICU admission. Results: Cox proportional hazards models, including a-STI, v-STI, VII, and RDRI as independent variables, returned a-STI as the only putative predictor for the development of AKI or severe AKI. An overall statistically significant difference (*p* < 0.001) was observed in the Kaplan–Meier plots for cumulative AKI events between patients with a-STI higher or equal than 0.37 and for cumulative severe AKI-3 between patients with a-STI higher or equal than 0.63. As assessed by the area under the receiver operating curves (ROC) curves, a-STI performed best in diagnosing any AKI and/or severe AKI-3. Positive correlations were found between a-STI and the N-terminal brain natriuretic peptide precursor (NT-pro BNP) (ρ = 0.442, *p* < 0.001), the sequential organ failure assessment (SOFA) score (ρ: 0.361, *p* = 0.004), and baseline serum creatinine (ρ: 0.529, *p* < 0.001). Conclusions: Critically ill patients who developed AKI had statistically significant different a-STI (on admission to ICU), v-STI, and VII than those who did not. Moreover, a-STI was associated with the development of AKI at day 5 and provided the best diagnostic accuracy for the diagnosis of any AKI or severe AKI compared with RDRI, VII, and v-STI.

## 1. Introduction

In the critical care setting, acute kidney injury (AKI) is frequent and associated with poor outcomes [1,2,3,4]. Currently serum creatinine (SCr) and urine volume are considered diagnostic criteria for AKI [5]. Nevertheless, oliguria is unspecific while the rise in SCr represent a late finding, occurring only when the glomerular filtration rate is severely impaired. Several biomarkers have been suggested in order to promptly identify AKI, but have never been widely adopted in clinical practice [6]. Therefore, as early renal replacement therapy (RRT) has been described to be associated with reduced mortality compared with the delayed or no initiation of RRT [7,8] but the diagnosis of AKI usually takes 12–24 h, an indicator for the prompt prediction of severe AKI within the first few hours of admission may trigger early treatments and obtain improved outcomes. 

Renal Doppler resistive index (RDRI) has been demonstrated to be able to predict AKI [9], distinguish between acute tubular necrosis and pre-renal azotemia [10], and mirror left ventricular systolic [11] and diastolic dysfunction [12], initial hypovolemia [13], and systemic tissue hypoxia [14] in order to establish the adequacy of systemic hemodynamics to avoid kidney hypoperfusion [15,16]. However, despite promising results, the RDRI and semi-quantitative evaluation of renal perfusion using color Doppler poorly predicted persistent AKI at day 3 [17], whereas RDRI is not currently recommended in the diagnosis or treatment of AKI due to its poor relationship with variations in renal blood flow [18]. The renal venous impedance index (VII) is a renal Doppler measurement to assess intrarenal venous flow, potentially able to reflect renal venous congestion. It was studied mainly in heart failure [19] and diabetic nephropathy [20], but no such studies have been performed in critically ill patients. The arterial systolic time intervals (a-STI) provide information concerning left ventricular function and the degree of adrenergic tone [21]. The venous time intervals (v-STI) have been suggested to be associated with intrarenal venous stasis and congestion [22].

This study aimed to explore the diagnostic accuracy of different Doppler parameters in the prediction of AKI in critically ill patients from a mixed intensive care unit.

## 2. Materials and Methods

This was a prospective, observational, exploratory study involving 63 critically ill patients admitted to ICU between 11 February 2021, and 11 July 2021. Patients were eligible for enrollment if they were older than 18 years of age and able to provide written informed consent. Exclusion criteria were included pregnancy, hemodynamic instability, history of chronic kidney disease requiring renal replacement therapy, arrhythmias, abdominal hypertension, obstructive uropathy, and positive SARS-CoV-2 nasopharyngeal swab. This study was approved by the institutional ethics committee (Comitato Etico Area Vasta Nord Ovest 19100/2021). All methods were performed in accordance with the relevant guidelines and regulations and informed consent was obtained from all patients.

### 2.1. Study Protocol

Participants were enrolled at ICU admittance; all underwent ultrasonographic (US) examinations and hemodynamic assessment as per clinical indications with the patient supine, using a 3.5-MHz convex probe for examination of the kidney and a sector-array probe with a 2–4-MHz transducer for examination of the heart. The treating physicians were blinded to the results of the renal ultrasound assessments. All patients were mechanically ventilated with 6–8 mL/kg tidal volume and peak end-expiratory pressure levels of 5 cmH_2_O. Ventilator settings were maintained at a constant setting in all patients during the examination. 

### 2.2. Color Doppler Ultrasonography

Intrarenal flow patterns—both interlobar arteries and veins were assessed at baseline—within 2 h from admission, by a single intensive care physician expert in Doppler ultrasonography with a GE Vivid S6 system (General Electric Healthcare Ultrasound System), during on-duty working hours, in order to avoid inter-observer variability. Measurements were acquired as previously described [13,14] and a simultaneous electrocardiographic trace (ECG-trace) was recoded in order to identify the phases of the cardiac cycle.

### 2.3. Calculated Parameters 

RDRI was calculated as the ratio of peak systolic velocity minus end-diastolic divided by the peak systolic velocity of the arterial trace.

VII was calculated as the peak maximum flow velocity minus the maximum flow velocity at nadir, divided by peak maximum flow velocity of the venous trace. Doppler waveforms were categorized into continuous and discontinuous flow patterns depending on whether or not the velocity at the nadir was greater than zero. When venous flow is discontinuous, the VI is 1.0, as the flow at nadir is 0. The VII therefore ranges from 0 to 1. 

a-STI indicates the proportion of the cardiac cycle during which there is an effective arterial perfusion and was calculated in milliseconds (ms) on the real-time ECG trace as (renal pre-ejection time/renal ejection time). Renal pre-ejection time (Rp-ET) was calculated from onset of QRS complex to the foot of renal Doppler waveform. Renal ejection time (RET) was measured from the foot of renal Doppler waveform to the dicrotic-notch of the renal pulse Doppler waveform. The higher the values, the lower the effective arterial perfusion. 

v-STI indicates the proportion of the cardiac cycle measured in ms from a QRS to the next QRS on the ECG trace during which there is no renal venous outlet flow and is calculated as follows: (cardiac cycle time–venous flow time/cardiac cycle time). v-STI represents the proportion of time during a cardiac cycle without venous outflow. The higher the values, the lower the venous return, indicating increased organ congestion. This variable also ranges from 0 to 1. When venous flow is continuous, the v-STI is 0, as the venous flow time equals the cardiac cycle time. 

**Figure 1:**
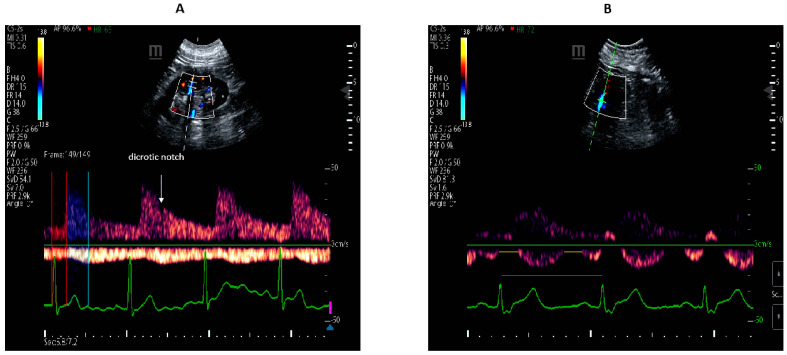
Panel (**A**) the arterial trace is shown above the green reference line. Renal Doppler resistive index is calculated as the ratio (S-D)/S, where S and D stand for peak systolic and end-diastolic velocities, respectively; arterial systolic time-intervals indicates the proportion of the cardiac-cycle during which there is an effective arterial perfusion and was calculated in milliseconds on the real-time ECG-trace as (renal pre-Ejection Time/renal Ejection Time). Renal pre-Ejection Time (red area) is calculated from onset of QRS complex to the foot of renal Doppler waveform. Renal ejection time (blue area) is measured from the foot of renal Doppler waveform to the dicrotic-notch of the renal pulse Doppler waveform; the continuous venous trace is shown below the green reference line. The venous impedence index is calculated as the peak maximum flow velocity minus the flow velocity at nadir, divided by peak maximum flow velocity. When venous flow is discontinuous, the index is 1.0, as the flow at nadir is 0. Panel (**B**) the discontinuous venous trace is shown below the green reference line. Venous systolic time-intervals indicates the proportion of the cardiac cycle measured in milliseconds from a QRS to the next QRS on the ECG-trace (pink line) during which there is no renal venous outlet flow (yellow lines) and is calculated as follows: (cardiac cycle time–venous flow time/cardiac cycle time). When venous flow is continuous, the v-STI is 0, as the venous flow time equals the cardiac cycle time.

RDRI, VII, a-STI, and v-STI are shown in Figure 1. 

Left ventricular ejection fraction and right heart systolic function inferred by tricuspid-anulus-plane-systolic-excursion (TAPSE) were also measured. Echocardiographic examinations were performed according to guidelines from the American Society of Echocardiography as recommended [23].

### 2.4. Clinical and Laboratory Data

Renal function was assessed daily according to the Kidney Disease Improving Global Outcomes (KDIGO) guidelines [5,24]. The lowest SCr recorded in the 3 months preceding trial inclusion in stable condition was considered the baseline value. The patients were subsequently assigned to one of three groups: patients without AKI, patients with any AKI (KIDIGO 1–3), and those who developed AKI-3 within 5 days. The following parameters were also recorded: sex, age, mean blood pressure (mmHg), heart rate (beat per minute), blood lactate level (mmol/L), standard base excess, arterial pH, gas-exchange, NT-pro BNP (pg/mL), SCr (mg/dL), continuous renal replacement therapy. NT-proBNP was measured using the Roche Diagnostics Cobas e analyzers chemiluminescent immunoassay E801. The reference range was <125 pg/mL. Plasma creatinine was measured in mg/dL by Roche Cobas C702 and total coefficient of variation (CV) was <2.06.

### 2.5. Statistical Analysis

Results were reported as median and interquartile (IQR) ranges, mean and standard deviation, or numbers and percentages (%) when appropriate. Categorical data were compared between groups by *X*^2^ test or Fisher exact test. The Shapiro–Wilk test was used to evaluate normal distribution of continuous variables. The Kruskal–Wallis non-parametric one-way analysis of variance (ANOVA) was used to compare variables between different KIDIGO stages. Cumulative probability for lack of development adverse outcomes was calculated with the Kaplan–Meier product limit estimator. The censored patients corresponded to the occurrence of AKI or AKI-3 complication. The log-rank (Mantel–Cox) test with pairwise comparisons was applied to evaluate the difference in probability for lack of complications. A Cox proportional hazards model with the studied ultrasonographic Doppler parameters was used to assess the risk of developing new-onset AKI within 5 days. Receiver operator characteristic (ROC) curves were plotted to examine the diagnostic accuracy of Doppler parameters in predicting the occurrence of any AKI or severe AKI. DeLong test was used to compare area under the curves (AUROCs). For each AUROC, the corresponding 95% confidence interval (CI) was calculated. Correlations between Doppler and clinical parameters were evaluated using non-parametric Spearman’s rank correlation. A sample size of at least 47 patients was required by assuming *r* = 0.4, power = 0.80, and *p* = 0.05. All tests were two-sided, and *p* values < 0.05 were considered statistically significant. Statistical analyses were performed by using IBM SPSS (version 27.0; Armonk, NY, USA), and R statistical environment (version 4.0.3, R Foundation for Statistical Computing, Vienna, Austria). 

## 3. Results

**Figure 2:**
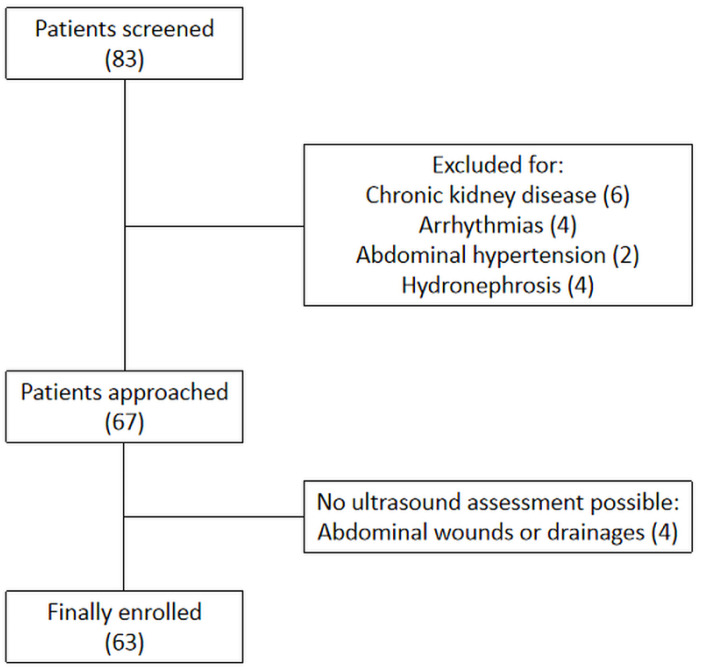
Flowchart of studied patients.

During the study period, 83 patients were screened. Baseline characteristic of patients are presented in Table 1. Among these patients, six were excluded due to the coexistence of chronic kidney disease, six were unsuitable due to arrhythmias or abdominal hypertension, four patients had a suboptimal acoustic window due to abdominal wounds or drainages, and another four due to unilateral or bilateral hydronephrosis (Figure 2). Thus, 63 patients were finally included: 36 with complicated elective major abdominal surgery, 10 with respiratory failure, nine with complicated thyroidectomy, three with sepsis, two with polytrauma, two with acute decompensated heart failure, and one with burns. According to AKI stages assessed on day 5, 35 patients (56%) developed AKI and 17 of them developed severe AKI (27%). Of the 35 patients with AKI, eight (13%) had AKI-1, 10 (16%) AKI-2, and 17 (27%) AKI-3. The diagnosis of AKI was made on day 1 for 10 patients, on day 2 for 17 patients, on day 3 for seven patients, and on day 4 for one patient only.

**Figure 3:**
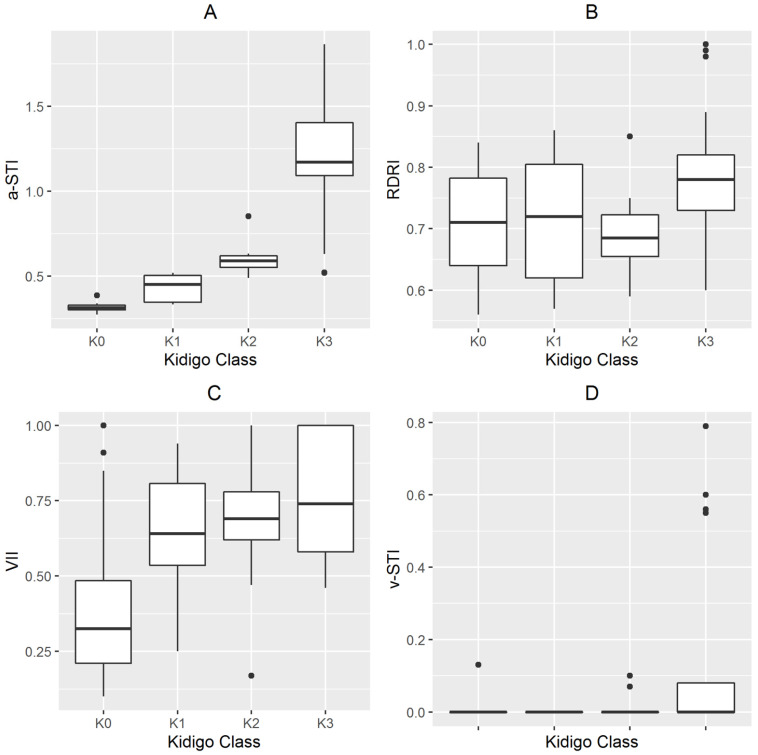
Doppler values assessed at ICU admission according to KDIGO stage at day 5. Panel (**A**) Arterial systolic time intervals (a-STI); Panel (**B**) renal Doppler resistive index (RDRI); Panel (**C**) renal venous impedence index (VII); Panel (**D**) venous time intervals (v-STI); Kidney Disease Improving Global Outcomes score (KIDIGO).

**Figure 4:**
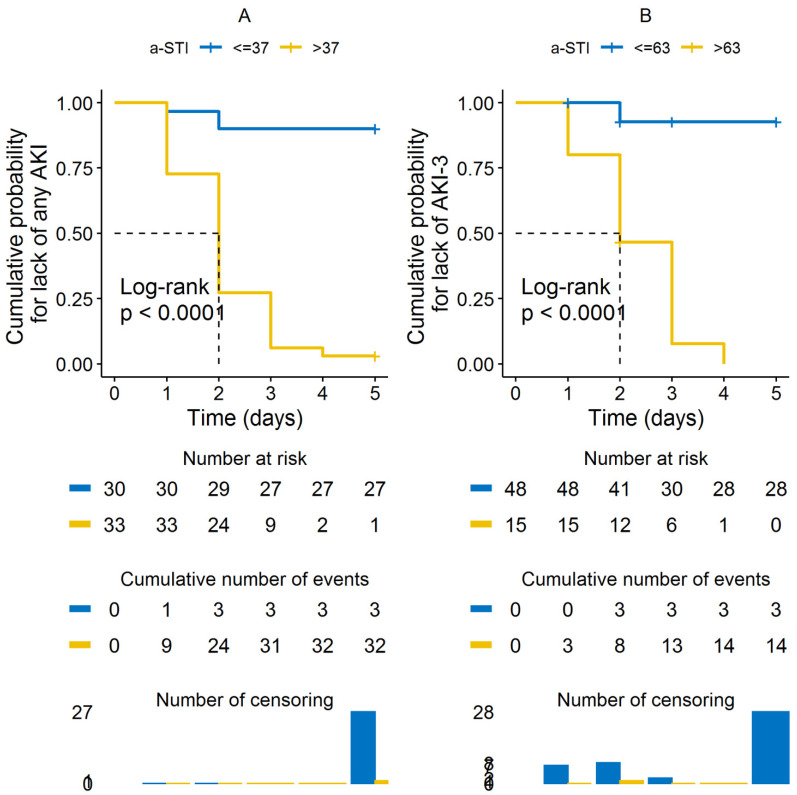
Panel (**A**): Comparison of the Kaplan-Meier curves for lack of adverse outcomes [any acute kidney injury (AKI)] in patients with a-STI equal or lower than 0.37 by using the log-rank test (*p* < 0.001). Panel (**B**): Comparison of the Kaplan-Meier curves for lack of adverse outcomes (AKI-3) in patients with a-STI equal or lower than 0.63 by using the log-rank test (*p* < 0.001). The number of patients at each time point is shown as a drop on the corresponding line. Patients were censored at day 5.

The patients’ characteristics and Doppler measurements according with different AKI stages are shown in Table 1 and Figure 3. Sequential organ failure assessment (SOFA) score, baseline SCr and brain natriuretic peptide, a-STI, v-STI, and VII were significantly different between groups (*p* < 0.05) (Table 1). A Cox proportional hazards model, including a-STI, v-STI, VII, and RDRI as independent variables, returned an overall *X*^2^ of 13.172 (*p* < 0.001), being a-STI the only variable associated with the development of AKI or severe AKI, (Table 2). An overall statistically significant difference (*p* < 0.001) was observed in the Kaplan–Meier plots for cumulative AKI events between patients with a-STI higher or equal than 0.37 (Figure 4A), and for cumulative severe AKI-3 between patients with a-STI higher or equal than 0.63 (Figure 4B).

### 3.1. Comparisons of Predictive Values for the Diagnosis of AKI

**Figure 5:**
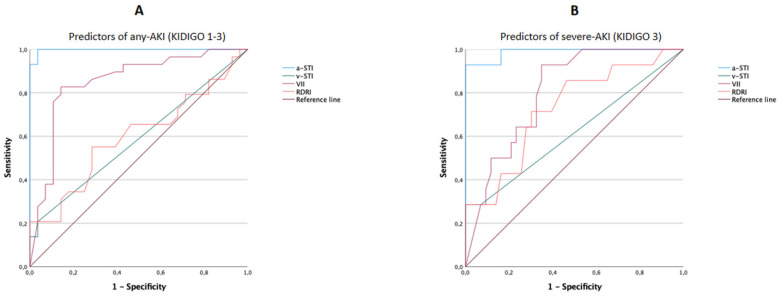
Receiver operator characteristic (ROC) curves for arterial systolic time intervals (a-STI), renal Doppler resistive index (RDRI), venous systolic time intervals (v-STI), venous impedence index (VII) as putative predictors for any acute kidney injury (AKI) (panel (**A**)) or severe AKI (panel (**B**)).

ROC curves were plotted to compare diagnostic accuracies and identify optimal cut-off values of a-STI, RDRI, v-STI, and VII in predicting any AKI or AKI-3. The area under the ROC curves of these indicators is shown in Table 3. As assessed by the AUROC, a-STI performed best either in diagnosing any AKI or severe AKI-3. Concerning the prediction of any AKI, the a-STI (AUROC: 0.994 (95% CI: 0.982–1.000); *p* < 0.001) was superior to VII (AUROC: 0.859 (95% CI: 0.758–0.960); *p* < 0.001), whereas RDRI (AUROC; 0.571 (95% CI: 0.429–0.713); *p* = 0.336) and v-STI (AUROC: 0.583 (95% CI: 0.442–0.723); *p* = 0.263) were not statistically significant (Figure 5A and Table 3). Concerning the prediction of severe AKI-3 the a-STI (AUROC: 0.985 (95% CI: 0.960–1.000); *p* < 0.001) was superior to VII (AUROC: 0.759 (95% CI: 0.640–0.878); *p* = 0.002) and RDRI (AUROC: 0.720 (95% CI: 0.574–0.577); *p* = 0.009), whereas v-STI (AUROC: 0.593 (95% CI: 0.418–0.768); *p* = 0.269) did not make a statistically significant contribution (Figure 5B and Table 3). 

### 3.2. Correlation Analysis of a-STI

We analyzed the correlations between a-STI and age, heart rate, mean artery pressure, gas-exchange, arterial lactate concentration, brain natriuretic peptide (NT-pro BNP), SCr, urinary output, ejection fraction, TAPSE, left ventricular ejection fraction, SOFA score, coronary heart disease, hypertension, or diabetes. Positive correlations were found between a-STI and NT-pro BNP (ρ = 0.442, *p* < 0.001), SOFA score (ρ: 0.361, *p* = 0.004), and baseline Scr (ρ: 0.529, *p* < 0.001). 

## 4. Discussion

The main findings of this study are that: (i) critically ill patients who developed AKI had statistically significant different a-STI, v-STI, and VII than those who did not; (ii) a-STI measured at ICU admission was predictive of development of AKI at day 5; (iii) a-STI provided the best diagnostic accuracy for the diagnosis of any AKI or severe AKI at day 5 compared with RDRI, VII, and v-STI.

Electrocardiographic time intervals derived from arterial RDRI waveforms are easy to perform, rapid, noninvasive, and repeatable. In our population, a-STI performed better than all other Doppler parameters. The technique is exactly the same as that used for sampling RDRI as it requires only a concurrent electrocardiographic trace on the screen to precisely identify the phases of the cardiac cycle and did not require additional equipment or software. a-STI differs from RDRI in that it is based on the analysis of the electrocardiographic cycle times, namely renal pre-ejection time (Rp-ET) and RET. These may provide additional useful insights in the adequacy of the global cardiovascular performance at the level of the kidney. We hypothesized that the better diagnostic accuracy of a-STI was due to changes in the electrocardiographic cycle times, occurring earlier as compared with changes in the arterial Doppler waveforms depicted by RDRI because the latter represent an expression of a hemodynamic disorder already in place, most of the time secondary to yet established parenchymal damage. Indeed, renal STIs are determined by the isovolumetric contraction time of the heart, electromechanical delay and the transmission of arterial pulse wave from the aortic valve to the interlobar arteries in the kidneys [25,26,27]. Hence, they summarize the net effect of cardiac systolic function, arterial stiffness, and venous compliance on the renal vascular system. Rp-ET may prolong owing to an impairment of cardiac contractility, decreased preload status, and increased afterload pressure or may shorten as a result of an increased preload status, decreased afterload pressure, or secondary to the use of positive inotropic agents. Pairwise renal-ET represent the period from the beginning to the finishing of stroke volume inferred at the level of interlobar arteries and its length is modified by preload conditions, strength of myocardial fibers, vasoactive agents and adrenergic activation [28,29,30]. In our population, v-STI was not associated to AKI probably because severe congestion with interrupted venous outflow was present only in a minority of patients (18%) and mainly in more severe stages, therefore they lose sensitivity in the less severe phases of congestion when the flow is still continuous even if pulsatile. On the contrary, VII seems to outperform v-STI in the prediction of AKI because it is able to detect the earliest forms of congestion characterized by pulsatile but still continuous flow, even if it is not able to accurately differentiate the severe forms in which the venous flow is already interrupted. Thus, VII seems to outperform compared to v-STI in the prompt detection of AKI secondary to initial congestion but is not accurate enough to promptly predict severe AKI secondary to the most severe stages of congestion. Moreover, our results allow speculation that congestion in critically ill patients represents only one among numerous determinants of AKI and probably not the most prevalent, unlike what happens in decompensated heart failure patients. In this setting, a-STI perform better probably because it is able to summarize in a single measurement either venous congestion or arterial hypoperfusion, suggesting that both can worsen kidney function, though kidney function may be maintained as long as renal perfusion is preserved. 

The mechanism of AKI is very complex and can be due to multiple causes, including but not limited to sepsis, metabolic disorders, ischemia-reperfusion injury, and hypoxia. 

Renal arterioles and veins may react differently depending on different etiologies of AKI. Our manuscript was not designed to investigate the different behavior of Doppler parameters according to different types of AKI and this issue needs to be further investigated. Moreover, the present study used the KDIGO criterion to identify patients with AKI. Nevertheless, we did not have patients with a clinical onset characterized by persistent oligo-anuria.

## 5. Conclusions

a-STI was associated with the development of AKI at day 5 and provided the best diagnostic accuracy for the diagnosis of any AKI or severe AKI compared with RDRI, VII, and v-STI.

Electrocardiographic time-interval waveforms derived from renal arterial Doppler resistive index are able to summarize in a single measurement either venous congestion or arterial hypoperfusion in critically ill patients.

## Figures and Tables

**Table 1:** Clinical characteristics, laboratory data and intrarenal Doppler parameters at ICU admission according to KDIGO stage at day 5.

Characteristics	All Patients *n* = 63	KDIGO 0 *n* = 28	KDIGO 1 *n* = 8	KDIGO 2 *n* = 10	KDIGO 3 *n* = 17	*p*
Male, *n* (%)	45 (71)	15 (54)	8 (100)	9 (90)	13 (77)	0.021 *
Age	70 (63–76)	65 (55–77)	74 (71–79)	71 (66–74)	71 (66–77)	0.147
BMI, kg/m^2^	25 (23–27)	25 (24–30)	25 (22–27)	24 (23–27)	26 (23–27)	0.302
Hypertension, *n* (%)	33	14 (50)	6 (75)	4 (40)	9 (53)	0.525
Diabetes, *n* (%)	11	4 (14)	1 (13)	0 (0)	6 (35)	0.112
CHD, *n* (%)	11	2 (7)	2 (25)	1 (10)	6 (35)	0.090
SOFA score	2 (0–5)	2 (2–2)	1 (1–2)	0 (0–1)	6 (0–8)	0.003 ^§^
HR (bpm)	70 (65–92)	70 (62–96)	69 (57–86)	83 (70–92)	70 (66–89)	0.752
MAP, mmHg	86 (67–94)	85 (70–98)	82 (67–92)	74 (63–97)	90 (69–94)	0.846
pH	7.40 (7.35–7.43)	7.41 (7.34–7.46)	7.41 (7.38–7.43)	7.42 (7.38–7.50)	7.40 (7.35–7.42)	0.777
pO_2,_ mmHg	108 (87–146)	100 (83–154)	100 (89–103)	109 (90–155)	108 (87–151)	0.968
pCO_2,_ mmHg	41 (40–45)	42 (40–49)	41 (40–44)	42 (34–47)	41 (38–45)	0.693
HCO_3,_ mmol/L	26 (23–28)	27 (24–28)	26 (26–30)	24 (21–28)	25 (23–27)	0.587
Base excess	1.4 (−1.9/3.9)	1.7 (−0.37/4.58)	1.8 (1.25/5.4)	−0.25 (−2.4/3.13)	0.9 (−2–20/2.40)	0.481
Arterial lactate, mmol/L	1.2 (1.0–2.2)	1.2 (0.9–2.2)	1.15 (1.03–1.53)	1.6 (0.98–3.28)	1.5 (1.00–2.50)	0.396
Serum K, mmol/L	3.8 (3.6–4.3)	3.8 (3.7–4.3)	3.7 (3.5–4.3)	3.8 (3.6–4)	3.7 (3.6–4.3)	0.585
Serum Na, mmol/L	136 (135–139)	136 (134–139)	136 (134–140)	139 (135–143)	136 (135–140)	0.128
SCr, mg/dL	0.99 (0.79–1.22)	0.81 (0.69–0.96)	1.13 (1.02–1.39)	1.15 (1–1.32)	1.16 (0.85–1.30)	<0.001 ^#^
NT-pro BNP, pg/mL	97 (35–532)	50 (33–108)	164 (20–407)	689 (35–1879)	1327 (85–2950)	<0.001 °
Urine output, mL/h	1700 (1200–2600)	1625 (1149–2902)	2090 (1348–3325)	2050 (1625–2565)	1540 (800–2420)	0.904
RDRI	0.73 (0.66–0.79)	0.71 (0.64–0.79)	0.77 (0.62–0.82)	0.69 (0.64–0.74)	0.78(0.73–0.86)	0.076
a-STI	0.42 (0.32–0.63)	0.31 (0.30–0.33)	0.45 (0.35–0.51)	0.59 (0.54–0.63)	1.17 (1.07–1.42)	<0.001 **
v-STI	0 (0–0)	0 (0–0)	0 (0–0)	0 (0–0.18)	0 (0–0.32)	0.026 ^§§^
VII	0.56 (0.31–0.79)	0.33 (0.21–0.52)	0.64 (0.47–0.84)	0.69 (0.58–0.84)	0.74 (0.57–1)	<0.001 ^##^
Venous flow pattern, Discontinuous *n* (%)	8 (13)	1 (4)	0 (0)	2 (20)	5 (29)	
Continuous *n* (%)	55 (87)	27 (96)	8 (100)	8 (80)	12 (71)	0.054
LVEF (%)	55 (48–66)	59 (49–68)	52 (49–59)	52 (45–64)	55 (43–66)	0.355
TAPSE (mm)	25 (22–28)	27 (22–29)	24 (21–28)	25 (22–28)	23 (20–27)	0.454
CRRT, *n* (%)	13 (21%)	1 (4)	0 (0)	1 (10)	11 (65)	<0.001

KDIGO: kidney disease improving global outcomes classification, *n*: numbers, AKI: acute kidney injury, BMI: body mass index, SOFA: Sequential Organ Failure Assessment, CHD: coronary heart disease, HR: heart rate, MAP: mean arterial pressure, pO_2_: arterial oxygen partial pressure, SCr: serum creatinine, NT-pro BNP: brain natriuretic peptide, RDRI: renal Doppler resistive index, a-STI: arterial systolic time intervals, v-STI: venous systolic time intervals, VII: venous impedence index, LVEF: left ventricular ejection fraction, TAPSE: tricuspidal anular plane systolic excursion, CRRT: continuous renal replacement therapy. Values are expressed as median and interquartile ranges (IQR), numbers and percentage. One-way analysis of variance (ANOVA) with the post-hoc Tukey-Kramer test was used to compare variables between different KDIGO stages (0–3). * *p* < 0.05 (KDIGO 0 vs. KDIGO 1), ^§^ *p* < 0.05 (KDIGO 2 vs. KDIGO 0, 1, 3 and KDIGO 3 vs. KDIGO 0), ^#^ *p* < 0.05 (KDIGO 0 vs. KDIGO 1, 2, 3), ° *p* < 0.05 (KDIGO 3 vs. KDIGO 0-1), ** *p* < 0.05 (KDIGO 0 vs. KDIGO 1, 2, 3; KDIGO 1 vs. KDIGO 2, 3; KDIGO 2 vs. KDIGO 3), ^§§^ *p* < 0.05 (KDIGO 0 vs. KDIGO 3), ^##^ *p*< 0.05 (KDIGO 0 vs. KDIGO 1, 2, 3).

**Table 2:** Univariate Cox proportional hazards model for the risk of any or severe acute kidney injury within the day 5 after ICU admission, by assuming as independent variables each Doppler parameter evaluated in this study.

**Any AKI**						
Doppler parameter	ß	SE	HR	95% CI		*p*
a-STI	0.779	0.370	2.180	1.055	4.504	0.035
RDRI	2.901	2.538	18.197	0.126	2630.557	0.253
v-STI	−1.374	1.010	0.253	0.035	1.830	0.173
VII	1.409	0.750	4.091	0.940	17.807	0.60
**Severe AKI**						
Doppler parameter	ß	SE	HR	95% CI		*p*
a-STI	2.599	0.675	13.477	3.580	50.507	<0.001
RDRI	2.901	2.538	18.197	0.126	2630.557	0.253
v-STI	−0.851	1.167	0.427	0.043	4.202	0.466
VII	0.184	1441	1.202	0.071	20.266	0.898

AKI: acute kidney injury; ß: regression coefficient; SE: standard error; HR: hazard ratio; CI: confidence intervals; a-STI: arterial systolic time intervals; RDRI: renal Doppler resistive index; v-STI: venous systolic time intervals; VII: venous impedence index.

**Table 3:** ROC curves related to any or severe acute kidney injury within the day 5 after ICU admission for each Doppler parameter evaluated in this study.

**Any AKI**								
Doppler parameter	AUROC	SE	*p*	95% CI		Cut-offs	S	Sp
a-STI	0.994	0.006	<0.001	0.982	1.000	0.37	0.91	0.96
RDRI	0.571	0.072	0.336	0.429	0.713	-	-	-
v-STI	0.583	0.072	0.263	0.442	0.723	-	-	-
VII	0.859	0.052	<0.001	0.758	0.960	0.55	0.86	0.86
**Severe AKI**								
Doppler parameter	AUROC	SE	*p*	95% CI		Cut-offs	S	Sp
a-STI	0.985	0.013	<0.001	0.960	1.000	0.63	0.94	0.96
RDRI	0.720	0.073	0.009	0.574	0.577	0.76	0.63	0.72
v-STI	0.593	0.089	0.269	0.418	0.768	-	-	-
VII	0.759	0.061	0.002	0.640	0.878	0.55	0.94	60

AKI: acute kidney injury; AUROC: area under the receiver operating characteristic curve; SE: standard error; CI: confidence intervals; S: sensitivity; Sp: specificity; a-STI: arterial systolic time intervals; RDRI: renal Doppler resistive index; v-STI: venous systolic time intervals; VII: venous impedance index.

## Data Availability

Data will be made available by the corresponding author for global collaboration on reasonable request, within the national restrictions imposed by privacy laws and ethics.

## Abbreviations

AKI	acute kidney injury
ICU	intensive care units
RDRI	renal Doppler resistive index
VII	venous impedance index
a-STI	arterial systolic time intervals
v-STI	venous systolic time intervals
Rp-ET	renal pre-ejection time
RET	renal ejection time
